# Processing and Testing of Reinforced PA66 Based Composites

**DOI:** 10.3390/ma14237299

**Published:** 2021-11-29

**Authors:** Alejandro Pereira, Alberto Tielas, Teresa Prado, Maria Fenollera, José Antonio Pérez

**Affiliations:** 1 Manufacturing Engineering Group (GEF) EEI Campus Lagoas, Universidade de Vigo (University of Vigo), 36310 Vigo, Spain; tprado@uvigo.es (T.P.); mfenollera@uvigo.es (M.F.); japerez@uvigo.es (J.A.P.); 2 Centro Tecnológico de la Automoción de Galicia (CTAG), Polígono Industrial a Granxa, 36475 Porriño, Spain; alberto.tielas@ctag.com

**Keywords:** PA66, PA66GF, weaves, reinforcement, overmoulding, composites

## Abstract

The new requirements in different sectors, such as aerospace, automotive and construction, for lightweight materials have led to an increase in demand for composite materials suitable for use in high rate production processes, such as plastic injection. This makes it necessary to look for matrices and reinforcements that, in addition to being compatible with each other, are also compatible with the injection process. It is in this area of research where the work presented here arises. To meet the two requirements mentioned above, this study contemplates a battery of composite materials obtained by combining PA66 and fiberglass, in different proportions and configuration, both for the preparation of the matrix and for reinforcement. For the elaboration of the matrix, two options have been evaluated, PA66 and PA66 reinforced at 35% with short glass fibre. To obtain reinforcement, six different options have been evaluated; two conventional fiberglass fabrics (each with different density) and four hybrid fabrics obtained from the previous ones by adding PA66 in different configurations (two over-stitched fabrics and two other fabrics). The different composite materials obtained were validated by means of the corresponding adhesion, peeling and resistance tests.

## 1. Introduction

### 1.1. Framework

Thermoplastics are sets of materials formed by polymers joined by intermolecular forces that form linear or branched structures. They become flexible or deformable at high temperatures, and can be melted and reformed several times. The demand for thermoplastic materials has had a great increase in the last years due to the increase in their possible applications [1], both in new products and in the replacement of materials, including metals. Their low cost, their good thermal and mechanical performance and their low specific weight have contributed greatly to this.

When designing thermoplastic composites, it is necessary consider some key factors. These materials are made of a thermoplastic matrix or binder and an immiscible reinforcement closely bounded to the binder. The composite properties depend mainly on the matrix, the reinforcement, and the adhesion between matrix and reinforcement. Reinforcement is the most influential parameter in mechanical properties. The matrix is the most important parameter in determining the other properties such as thermal behaviour, durability, chemical and fire resistance. Finally, the reinforcement/matrix adhesion is essential for the final properties [2].

The main reinforcements used are fibres, foams, flat materials and nanofillers. The utilization of fibres in the fabrication of composites has revealed significant applications in a variety of fields such as aerospace, automotive and construction [3,4]. Fibres addition increases the mechanical properties, improves creep behaviour, can cause some anisotropy according to the fibre orientation that can lead to different shrinkage in different directions, and increases viscosity, so makes the processing more difficult [5].

The principal fibre parameters to take into account in composite processing are: fibre nature, addition level, real fibre sizes, homogeneity of the fibre distribution, and sizing, which governs the fibre/matrix adhesion [6]. Of the available fibres, natural and synthetic, the glass fibre (GF) is the most common for polymeric matrix composites, accounting for 95%, because it offers excellent strength durability, thermal stability, impact, chemical and friction resistance, wear properties and low cost. Less frequently used are carbon, basalt and aramid [7]. There is a practical limit of about 70 volume percent reinforcement that can be added to form a composite; at higher percentages, there is too little matrix to support the fibres effectively.

An additional issue to consider is whether the properties of composites vary depending on the fibre. When the properties of the composite vary with the length of the fibre it is called discontinuous or short fibre. These types of material present random or preferred orientation of fibres. On the other hand, if any further increase in length does not further increase, the elastic modulus, the composite is considered continuous or long fibre, and features unidirectional or bidirectional orientation of fibres.

Different fibre forms can be used to strengthen. Spun filaments are assembled, with technologies similar to textile fabrics, into strands, threads and rovings that can be woven or knitted. There is a variety available: chopped or milled fibres, strands, yarns (plied or cabled), texturized and volumized products, mats and prepregs. The reinforcing structures and fabrics are characterized by the woven pattern or crossing scheme of the warp (lengthwise) and weft (perpendicular to the warp) yarns, the count or number per centimetre of warp and weft yarns, and the yarn types [8].

Hybrid composites are structures consisting of more than one type of fibre. As investigated, there are several ways to optimize composites by varying fibre content, its orientation, size, or manufacturing processes. Recent research develops hybrid compounds combining natural and synthetic fibres. In some of them, certain manufacturing defects are evidenced such as misalignment, waviness, fibre breakage, fibre/matrix debonding, delamination and voids in the matrix. These defects minimize the expected improvement in mechanical properties [9].

For fibre-reinforced composites (FRC), the matrix performs different functions. First, it binds the fibres together and is the medium by which the external stress applied to the fibres is transmitted and distributed, such that only a small proportion of the applied load is sustained by the matrix. Ductility and much lower elastic modulus than that of the matrix are recommended. The second function is to protect individual fibres from surface damage because of mechanical abrasion or chemical reactions with the environment. The matrix also serves as a barrier to the crack propagation [10].

Among all FRC; with polymer, metal, ceramic or carbon matrix; the polymers are the most commonly used today for their light weight, high stiffness and strength, as well as versatility and ease of manufacture [11,12]. The selection of matrix material is often influenced by the required temperature performance of composite; polymers are usually selected for lower temperature, up to 250 °C; and ceramics are used in high-temperature applications. Polyamide 66 (PA66) has attracted attention as a resin matrix because of its excellent melt flowability, good processability and mechanical properties [13].

Strengthening matrix reinforcement interphases has been the focus of a great amount of research, particularly in thermoplastic composite materials. Compatibility can be increased by fibre surface treatment, changes in the polymer matrix, or both. Among the compatibilising methodologies, the most widely used is fibre treatment with coupling agents and matrix modification techniques, such as alkali treatment, acetylation and graft copolymerization [14].

FRC processing involves manufacturing of fibre preforms and then reinforcing these fibres with the matrix material using various techniques. The over-moulding process offers manufacturing in short cycle times and to combine the characteristics of two or more polymeric materials in a single mould without mechanical interlocking or adhesive bonding. Over-moulding process is one of the growing advanced technologies for fabricating lightweight composite structures used in the aerospace, automotive and construction industries [15,16].

### 1.2. Background

Under the technical framework exposed on FRC processing, several research has established the basis of the present investigation. Asadi et al. [17] studied reinforced textile membranes of PVC (polyvinylchloride)-coated PET (polyethylene terephthalate) fabric to replace the traditional reinforcement of building structures under various criteria, such as mechanical properties, insulation, light transmission, fire retardation, folding capacity and cost.

Mikolajczyk et al. [18], investigated building composite beams made from Mapei Mapefill concrete reinforced with knitted mesh, fabricated using the technology of warp knitted fabrics. Three variants of knitted technical meshes made of polyamide PA6, PP (polypropylene) and GF threads were used as reinforcement. Regarding the mechanical properties, in case of the composite with GF, strength properties were twice better compared to the original concrete beam.

Textiles in prefabrication as well as in the retrofitting of existing concrete or masonry structures were studied by Koutas et al. [19]. Fibre rovings arranged in two or more directions were used. Textiles with polymers improve the stability of the textile material and the mechanical interlock between the textile and the matrix. As a result, the stiffness, the ultimate flexural or shear capacity, and the performance under serviceability loads are increased. In addition, cracking is better controlled.

Franke et al. [20], carried out composite construction parts, specifically sandwich materials, with good load-bearing characteristics. Composites of pure PA66 and PTFE (polytetrafluoroethylene), PTFE-PA66 compounds with a PA66 injection matrix and GF reinforced core were developed, resulting in compounds with high mechanical strength.

Fiorotto’s team [21], from the University of Padua, has investigated the manufacturing of a thermoplastic with a reinforcement sheet of a thermoformed fibre, which is inserted in the mould and, right after the thermoplastic is injected. It produces an economic composite with good technical qualities. Adhesion parameters between the fibre and the polymer have been investigated experimentally.

Khondker’s team has experimented with the injection moulding with textile inserts of knitted polyethylene (PE) fabric as reinforcement and only PE as matrix [22]. Since they are similar elements, there would be a high bond between matrix and reinforcements. In addition, an impregnation of a resin in the textile was applied. As a result, they found that the tensile properties were dependent on the impregnated resin.

Yang et al. investigated the impact of PP composites, reinforced with fibres of the same PP, and of PP reinforced with knitted GF [23]. Panels of these composites are made using the injection-compression moulding technology, studying the behaviour as a function of the composites’ temperatures. It was demonstrated that the impact strength of PP/PP and GF/PP was very similar.

In the University of Zaragoza [24], the injection on fabric has been investigated from the aesthetic point of view, studying the injection pressure parameters inside the mould in order to achieve a good final finish. The methodology to determine the relationship be-tween pressure and flow has been studied, with the aim to optimize this process. For this purpose, they have designed a spiral mould with pressure sensors and three types of textiles with different qualities and different foam thicknesses.

The German company “BASF” has developed a material called “Ultramid Structure^®^” [25]. It is a long GF-reinforced polyamide (PA) in the form of sheets. This product represents a significant advance in performance. The exceptional feature of the composites manufactured with long glass fibre-reinforced plastic components is the 3D GF net that they form during the conventional injection moulding, which gives the product exceptional physical properties, both at high and low temperatures. The fibre net forms the skeleton of the component, which lasts even after calcination. This structure is the reason why warping, plastic deformation behaviour and energy absorption in this type of material have a performance close to metals.

The present research focuses on developing a lightweight thermoplastic reinforced composite with high mechanical properties, meeting the requirements of the aerospace, automotive and construction sector. As previously shown, there are several investigations in the field of addition of different elements such as natural and non-natural fibre-reinforcements with varied matrixes. However, the significance of this paper is based on the use of over-moulding technology for composites manufacturing. The composites are made of PA matrixes reinforced with PA and fibre glass fabrics, which combine the high specific strength and stiffness of the continuous fibres with the design freedom and flexibility of short fibre. One of the great challenges is to acquire a good union between the matrix and the fabrics. To solve this problem, two different manufacturing techniques are presented: Over-stitching of PA thread on fibre glass fabric; and manufacturing by weaving of hybrid fabrics, with GF, on a prototype loom. Adhesion, peeling and resistance test are carried out to validate the properties of the new composites.

## 2. Materials and Methods

### 2.1. Introduction

**Figure 1:**
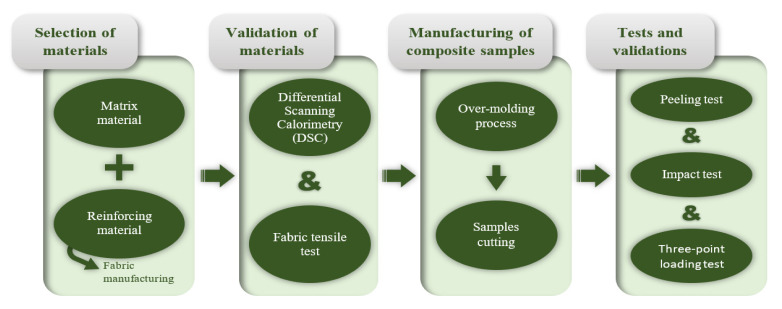
Experimental procedure.

This section is organized based on the phases shown in Figure 1 that also follow the line of investigation for the achievement of the reinforced composite material. First, the materials for the matrix and the reinforcing ones are selected, the latter being manufactured in the form of fabric. In order to corroborate the material compatibility between the matrix and the reinforcing materials, their melting points are validated by a differential scanning calorimetry test. Moreover, to verify if the manufacturing process damaged the fabrics, a tensile test of the manufactured fabrics is performed. After that, the composite samples are manufactured, by injection over-moulding, combining the matrix and reinforcement materials. Finally, these samples are tested to validate their properties.

### 2.2. Materials

Two types of materials should be selected as discussed above, the matrix and the reinforcement materials. These materials must be chemically compatible, so that the matrix helps to stiffen the fabric.

#### 2.2.1. Matrix Materials

PP and PA are the most widely introduced thermoplastics in the automotive sector [4,26]. The first ones are used for general parts while the second ones are used for parts with more restrictive mechanical requirements. Since the goal of this work is to develop a thermoplastic composite material with high mechanical properties, PA is finally the selected material.

After an exhaustive benchmarking analysis of commercial PA, and weighting their mechanical, physical and thermal properties, two PA66 have been chosen as the base matrix material, whose properties are shown in Table 1:

- Zytel^®^ 70G35HSL NC010 reinforced with 35% short GF (PAGF).
- Zytel^®^ 101L NC010 non reinforced (PA).

#### 2.2.2. Reinforcement Materials

From the side of the fabrics, and due to the lack of suppliers in the market for hybrid fiberglass fabric with PA, this material was obtained by manufacturing processes. For the manufacture of the hybrid fabrics, two different techniques were studied:

1. Over-stitching manufacturing of PA thread on fiberglass fabric.
2. Manufacture by weaving of hybrid fabrics, with fiberglass on a prototype loom.

##### Over-Stitching Manufacturing

To produce fabric by over-stitching, it is necessary to select the weight and orientation of the fabric fibres since these features affect the greater or lesser opening of the mesh.

The more open the mesh, the better the embedding of the thermoplastic in the fabric and consequently the better the adhesion at the matrix-fibre interface.

Regarding the orientation of the fibres in the fabric, a multidirectional fabric (0°/90°) was chosen to minimize the anisotropy of the properties of the composites.

Keeping these orientations as a basis, fabrics with lower weights were selected for processing since they have a bigger pitch between threads. Two fiberglass fabrics with weights of 86 and 125 g/m^2^ with different mesh opening were selected to manufacture by over-stitching (Table 2):

- Fiberglass of 86 g/m^2^ (GF86).
- Fiberglass of 125 g/m^2^ (GF125).

**Figure 2:**
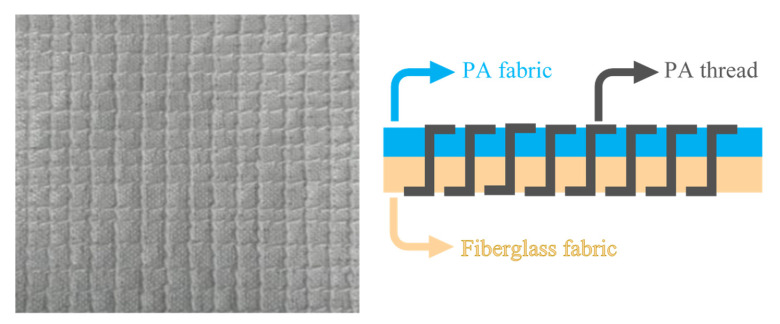
Hybrid fabric constitution.

To facilitate the ulterior injection over-moulding process, a PA fabric that acts as a coupling agent between the fiberglass fabric and the matrix, was added. To stitch both fabrics a PA thread was used (Figure 2). Both PA fabric and PA thread are made of PA66, the same material as the base, which ensures the chemical compatibility and helps to stiffen the fiberglass fabric in the process of over-stitching.

Characteristics of the PA fabric and PA thread are shown in Table 3.

As a result of the over-stitching process two hybrid fabrics are obtained:

- Fiberglass + PA thread by over-stitching of 86 g/m^2^ (PAGF86).
- Fiberglass + PA thread by over-stitching of 125 g/m^2^ (PAGF125).

##### Weaving Manufacturing

**Figure 3:**
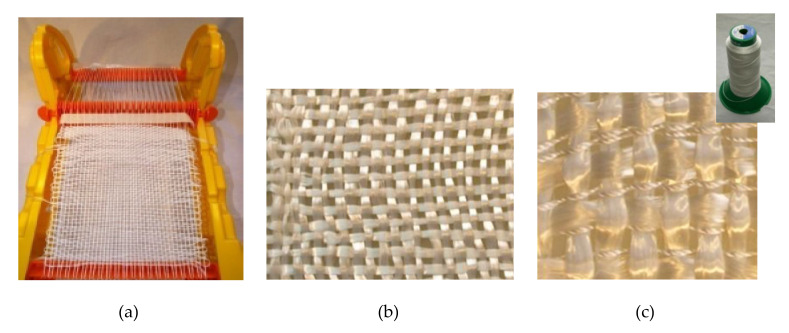
(**a**) Prototype loom. (**b**) Fiberglass fabric. (**c**) Fiberglass + PA hybrid fabric.

For the fabrics manufactured with fiberglass on a prototype loom (Figure 3a), a single-end roving for long fibre thermoplastics PA composites SE 4535 from 3B Fibreglass was selected (Table 4). The proprietary sizing chemistry is specifically engineered to provide excellent wet out, optimum resin-fibre load transfer and adhesion for exceptional mechanical performances.

Figure 3b shows the fiberglass fabric manufactured. Another hybrid fabric composed by fiberglass roving with PA thread was also created (Figure 3c). The procedure for making this hybrid fabric is the same as for the fabric made with only fiberglass, with the difference that, in this case, for each horizontal fiberglass thread two PA66 threads are interspersed.

These two fiberglass fabrics were functionalized with Aminopropyltriethoxysilane (APS) to provide them with new properties [27,28], obtaining the following fabrics to the tests:

- Functionalized fiberglass (GFF).
- Functionalized fiberglass + PA thread (GFFPA).

#### 2.2.3. Validation of Materials

Before manufacturing the composites, differential scanning calorimetry (DSC) tests were performed in order to validate the melting point of the thermoplastic materials referred in the manufacturers’ datasheet (fabric, textile thread and granules for the injection) and to establish a correlation between the melting points of the PA threads and fabrics and the PA pellets to be injected. The tests were carried out on a previously calibrated “DSC Perkinelmer precisely” equipment, according to specifications of the standard ISO 11357-1:2017 [29]. Three samples of each material (PA66 fabric, PA66 thread, PA matrix and PAGF matrix) were tested resulting in 12 tests.

Once the fabrics were manufactured, to assess whether the manufacturing process damaged the fabrics, a tensile test on these fabrics was performed on an Instron 4505 universal testing machine at room temperature (23 °C) with a 1KN load cell and a constant speed of 50 mm/min until failure, according to specifications of the standard ISO 527-4:1997 [30]. These processing conditions have a direct influence on the morphological structure of the parts [31,32], and this structure, in turn, influences the properties of the material [33]. This test was not performed with functionalized/hybrid fabrics because no comparison values are available. With this test it was sought that the maximum force supported by the hybrid fabric was at least equal to or greater than the force supported by the GF/PA fabric. Then, three samples of each material (PA66 fabric, PA66 thread, GF86, GF125, PAGF86 and PAGF125) were tested resulting in 18 tests.

### 2.3. Methods

#### 2.3.1. Manufacturing of Composite Samples by Over-Moulding Process

**Figure 4:**
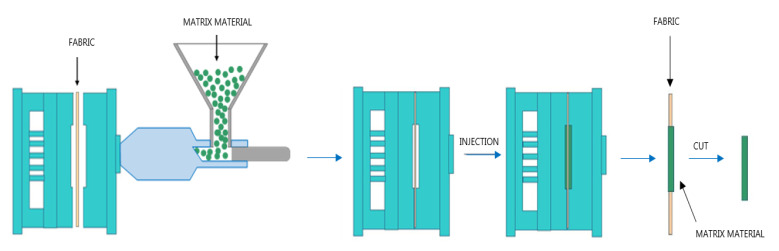
Injection over-moulding process sketch.

In the manufacturing of composite samples, the injection over-moulding process has been chosen. In this process, a piece of fabric is placed between the plates of the mould and a matrix material is injected to reinforce the material (Figure 4).

For the injection, an Engel 350 ton injection mould machine with a 60 mm diameter screw and a mould with flat plates was used. Both the equipment and the mould are not adapted for injection by the injection-compression process. The mould is also not prepared for the manufacturing of samples by a multipoint or sequential injection process, consequently it was not possible to apply these techniques in order to reduce the injection pressure during the process. The fabric was placed directly in the cavity of the movable plate. It was fixed by a double-faced tape at each of the corners and in the middle of the fabric longitudinal distance in order to ensure the total support of the fabric.

Fourteen samples were injected considering the different combinations of matrix and reinforcement materials, corresponding to the design of experiments that is shown in Table 5.

**Figure 5:**
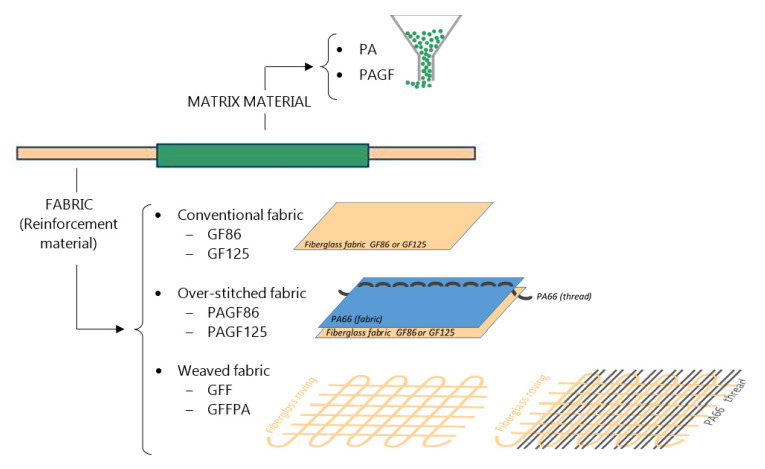
Explanatory diagram of the composition of the samples.

Figure 5 shows an explanatory diagram of the samples’ composition.

The processing conditions were selected following the manufacturer’s recommendations, which essentially depend on the matrix material. Table 6 shows these conditions. Due to the typical hygroscopicity of PA, it was necessary to perform a drying process prior to the injection process, which was carried out in a Koch Technik KKTT55 dehumidifier at 80 °C for 4 h, as recommended by the manufacturer.

Sample preparation plays a major role in recorded test results. Depending on the geometry and the type of test, different shapes are proposed for cutting the 3 types of samples [34]. For the tensile and peeling tests, rectangular samples of 25 × 100 mm^2^ were prepared. In the case of the three-point loading test, 100 mm diameter circular samples were cut, and for the impact test the samples had a 60 mm square shape.

#### 2.3.2. Tests and Validations

**Figure 6:**
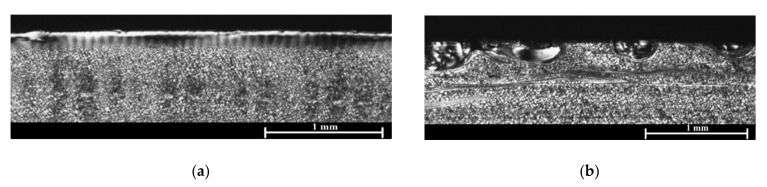
Morphological structure. (**a**) Injection side. (**b**) Ejector side.

The morphological structure influences the properties of the material, so before realizing the tests in the different samples to evaluate the composites, the shell-core-shell type morphology of them due to the injection process was evaluated. In Figure 6 it can be observed the asymmetry in the morphology resulting from the difference in the cooling velocity on the side of the fabric with respect to the side without fabric. On the injection side (side without fabric), due to the better thermal conductivity of the mould insert material, the cooling velocity is higher, which causes a well-defined layer. On the other hand, on the ejector side (side with fabric) due to the low thermal conductivity of the fabric, the cooling velocity is much lower than the injection side one, leading to a decrease of the shell advantaging the nucleus formation [35].

In order to evaluate the function of the diverse fabrics used and final reinforced composite, numerous tests were performed. Table 7 summarizes all the tests performed to verify each property.

**Figure 7:**
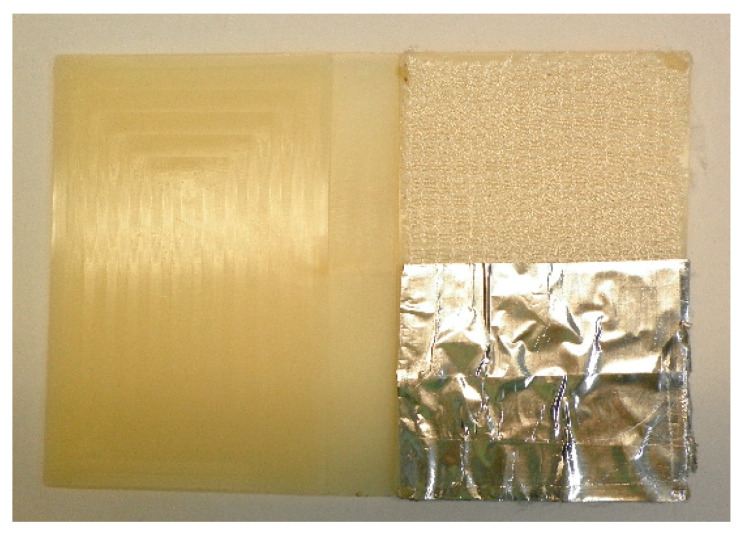
Prototype part for the manufacturing of the peeling test sample.

Once the composites have been manufactured by injection, three tests are conducted to assess their behaviour. To evaluate the adhesion of the fabrics to the matrix, the peeling test was performed according to ISO 11339 [36]. Tests were carried out on the previous universal testing machine at room temperature (23 °C) with a 1 kN load cell and a constant speed of 50 mm/min. Three samples of each composite (PA-86, PA-125, PAGF-86, PAGF-125, PA-PA86, PA-PA125, PA-F, PA-FPA, PAGF-PA86, PAGF-PA125, PAGF-F and PAGF-FPA) were tested resulting in 36 tests. For the manufacturing of the samples for the peeling test, a part of the plate was covered with aluminium adhesive tape, to avoid adhesion and to be able to use that area for pulling, as shown in Figure 7.

**Figure 8:**
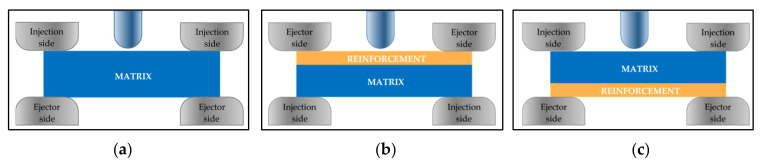
(**a**) Sample without fabric reinforcement. (**b**) Reinforced sample, ejector side. (**c**) Reinforced sample, injection side.

To determine the influence of the hybrid fabric on the impact properties with respect to the initial matrix, impact tests were carried out in a Fractovis Plus machine using a drop weight instrument at room temperature (23 °C) with a weight of 5.045 kg, a dart with a 20 mm diameter ball tip, dropping from a 1 m height [37,38]. Samples with fabrics (without reinforcement) and samples with the hybrid fabric were tested. The composites were tested with the face of the fabric under impact and with the face of the matrix under impact as shown in Figure 8, due to the different morphological structures as commented before. Three samples of each composite at both sides (PA-PA86, PA-PA125, PA-F, PA-FPA, PAGF-PA86 and PAGF-PA125) and three samples of the two-matrix material (PA and PAGF) were tested resulting in 42 tests. PAGF-F and PAGF-FPA were not tested because of poor adhesion between matrix and fabric.

The three-point flexural test used was the one proposed by Nunes et al. [39] and Okereke [40], where the sample is supported in three uniformly distributed points inserted in a circumference with a diameter of 9.35 cm. The load is applied in the centre with constant speed, in the same way as in the dart drop impact test. The composites were tested positioned on both sides and following the work of Wakeman’s team [41]. Tests were carried out on the same universal testing machine at room temperature (23 °C) with a 1 KN load cell and a constant speed of 5 mm/min up to 2 mm maximum deformation. Three samples of each composite at both sides (PA-PA86, PA-PA125, PA-F, PA-FPA, PAGF-PA86, PAGF-PA125, PAGF-F and PAGF-FPA) and three samples of the two-matrix material (PA and PAGF) were tested for each load orientation, resulting in 30 tests.

## 3. Results and Discussion

### 3.1. Differential Scanning Calorimetry (DSC) Tests

In the different DSC tests, it was checked that the melting temperature, experimentally measured in the samples, corresponds to that shown by the manufacturers in their datasheets, with variations under 0.5%. It was also verified that all the materials have similar melting temperatures (Table 8), the largest difference being 5.5 °C between the PA66 textile thread and the PA matrix material, which shows the stability of these materials and the stability of the transitions.

### 3.2. Tensile Test

**Figure 9:**
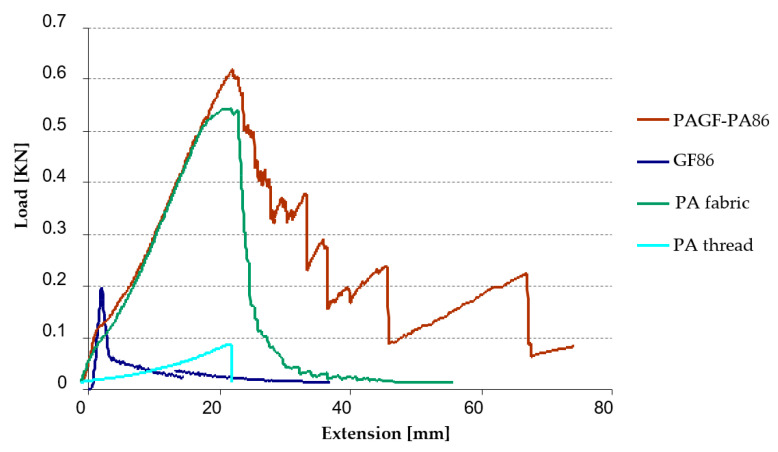
Tensile test curve of PA86 hybrid fabric compared with its components.

As an example of the graphs obtained from the tensile tests, Figure 9 shows the graph Load vs. Extension of the composite number 11, according to the design of experiments (Table 5). This graph shows the composite PAGF-PA86 compared with its component materials (GF86, PA fabric and PA thread).

The graph shows that the maximum force supported by the composite PAGF-PA86 is greater than its components. In Table 9 the values of maximum force obtained for all the material are resumed. It can be observed that the tensile strength of GF125 fabric is higher compared to the tensile strength of the GF86 fabric. In composites this difference does not exist, assuming similar values. This fact is justified by the use of the PA fabric with short fibre in the manufacture of the composite, since the value of the maximum force recorded for the composite practically corresponds to the value obtained in the PA66 fabric test. It means that the manufacturing process by industrial over-stitching does not influence on the properties of the composites, thus guaranteeing the viability of the manufacturing process.

Another cause of this behaviour could be the processing conditions that influence the morphological structure of the parts [42,43], and the type of morphological structure influences the material properties [44]. The injection moulding process, as discussed above, induces a shell-core-shell type morphology. The unbalance in the cooling rate causes an asymmetry in the morphological structure that can increase the temperature gradient along the composite thickness [45].

### 3.3. Peeling Test

Table 10 shows the mean peel strength and deviation of the composites tested, according to the design of experiments (Table 5). During the tests it was revealed that in the samples reinforced with conventional fiberglass fabric, the fabric is evenly pulled off. However, in the samples based on hybrid fabrics, elaborated both through sewing and weaved by a weaving machine, there are areas in which the adhesion is higher than the reinforcement, thus when performing the test, the fabric is stretched until it breaks.

From these results it can be observed that the adhesion load is higher for the samples with matrix without fiberglass (PA matrix) than that of the filled ones (PAGF matrix). The best results in peeling test correspond to composites 3, 7 and 8, all of them with PA matrix. This could be due to any kind of chemical incompatibility between the matrix filled with GF and the reinforcement, apart from the denser resin, which significantly worsens the impregnation of the reinforcement.

**Figure 10:**
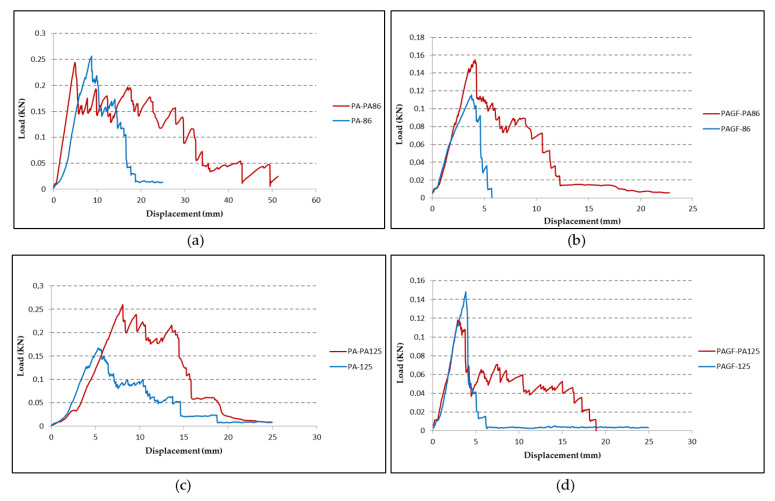
Comparison of peeling test results between conventional fiberglass fabrics and hybrid fabrics. (**a**) PA matrix + GF86, composites 7, 3. (**b**) PAGF matrix + GF86, composites 11, 5. (**c**) PA matrix + GF125, composites 8, 4. (**d**) PAGF matrix + GF125, composites 12, 6.

However, analysing in more detail the graphs of Figure 10 that compare the results of the samples with hybrid fabrics with those with conventional fiberglass fabrics, it can be observed that although the mean peel strengths are similar, the adhesiveness remains longer.

**Figure 11:**
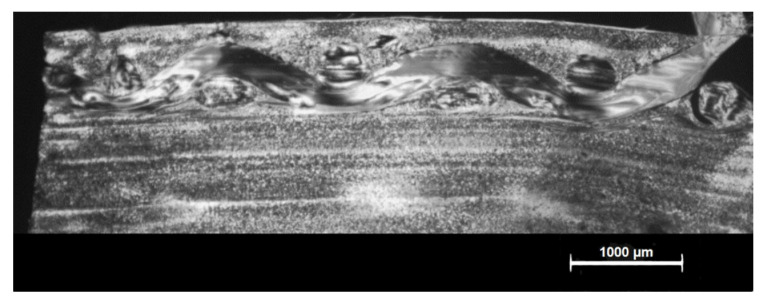
Impregnation of the PA thread in the PA matrix.

It can be observed in Figure 11 the total impregnation of the PA thread in the matrix, which confirms that PA thread improves the adhesion between the reinforcement and the matrix, in case of composites 7 (PA-PA86) and composite 8 (PA-PA125).

The samples with reinforced matrix material and functionalized fiberglass reinforcement fabrics (PAGF-F and PAGF-FPA) finally did not undergo peeling test because the fabric could detach simply pulling by hand. On the other hand, the samples with woven fabric to reinforce the PA66 matrix are the ones that obtained the worst results. Based on these results and given the difficulty and slowness of making these fabrics, it is considered that the properties of samples with woven fabrics will no longer be evaluated.

It would be interesting, in future work, to test other types of fabrics to improve the adhesion. Working for example with binders could ameliorate adhesion, controlling the peel strength by different kinds of binder layer formations and also by the different binder-fibre interaction [46]. Reactive binders could offer the potential to provide much larger interplay adhesions, as demonstrated in the study of glass/vinyl ester composites [47].

### 3.4. Impact Test

**Figure 12:**
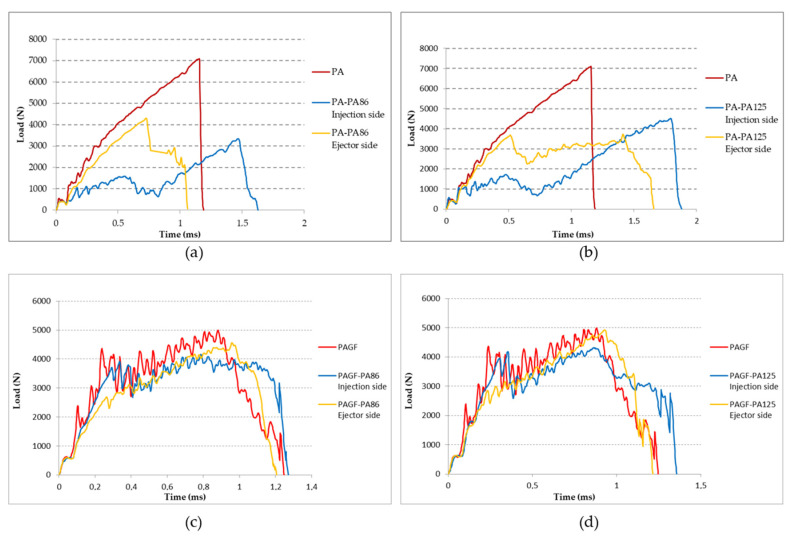
Comparison of the impact test according to the impact side on the composite sample and with respect to the matrix material without reinforcement. (**a**) PA matrix and PA-PA86 composite. (**b**) PA matrix and PA-PA125 composite. (**c**) PAGF matrix and PAGF-PA86 composite. **(d**) PAGF matrix and PAGF-PA125 composite.

As described in test and validations, the impact test was performed on both sides of the composites and on the matrix samples without fabric reinforcement. The results of these tests are shown in Figure 12. In each graph, the behaviour on each side of the composite sample is compared to the sample of the matrix material (red line). It was observed that in the case of the PA matrix (Figure 12a,b), the behaviour of the failure load composite is lower than the failure load matrix. However, the energy absorption is higher. Furthermore, since the impact on the side of the fabric reinforcement (yellow line) is produced on a more rigid area, the rigidity is higher than when the impact occurs on the matrix side (blue line). In the latter case, a ductile behaviour is more present at the beginning, with a longer duration of the impact, and increases the capacity of energy absorption. This behaviour is interesting but does not improve the failure load matrix material. However, in the case of PAGF matrix (Figure 12c,d), the working of the composite is similar to that of the matrix, mainly when the impact is on the side of the reinforcement.

**Figure 13:**
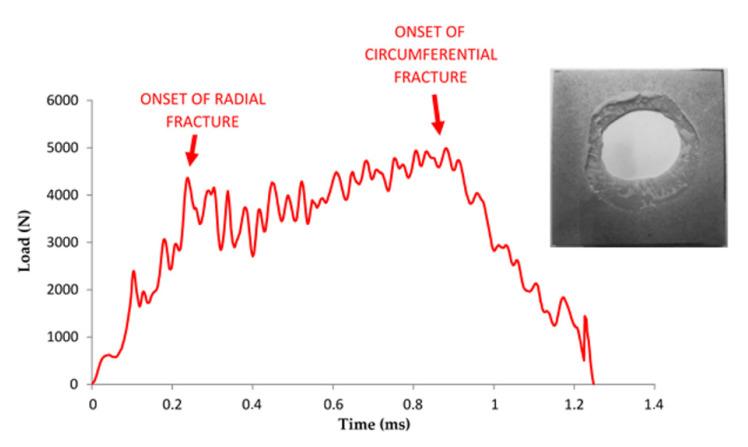
Correlation between fracture propagation and the Load vs. Time for PAGF matrix material.

According to Silva et al. [48], the mechanism of crack initiation and propagation was found to have a strong impact on the failure mode induced in the structure. They distinguished four failure modes: Mode I; progressive crashing with microfragmentation and delamination with a good energy absorption. Mode II, brittle fracture with large fragmentation. This fracture mode corresponds to unstable and catastrophic failure of the sample. Mode III, brittle fracture with progressive crashing and medium fragmentation, and Mode IV, progressive folding with mushrooming effect. The case of matrix PA and PAGF are Mode II of failure. It was observed on the graphs that PA matrix material registers only radial fracture while PAGF matrix material reveals both radial and circumferential fracture (Figure 13). This is due to the greater rigidity and anisotropy of PAGF compared to PA.

**Figure 14:**
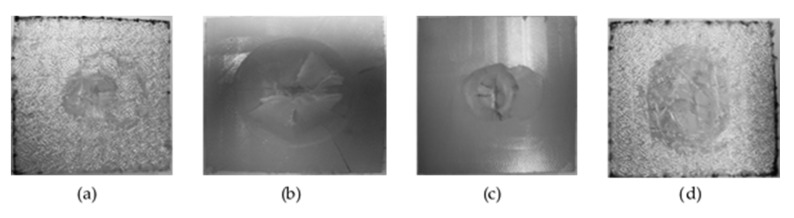
Impact samples on PA-PA86 composite. (**a**) Impact face on sample with ejector side impact. (**b**) Opposite impact face on sample with ejector side impact. (**c**) Impact face on sample with injection side impact. (**d**) Opposite impact face on sample with injection side impact.

**Figure 15:**
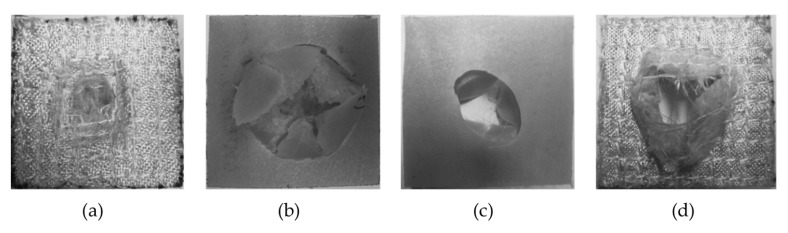
Impact samples on PAGF-PA86 composite. (**a**) Impact face on sample with ejector side impact. (**b**) Opposite impact face on sample with ejector side impact. (**c**) Impact face on sample with injection side impact. (**d**) Opposite impact face on sample with injection side impact.

If both sides of the impact on PA-PA86 samples are analysed, it is observed that on the opposite side of the impact the fractures are developed in a radial direction, which corresponds to a radial fracture evidenced by the curve registered (yellow line in Figure 12a). A well-defined whitish circle is observed in Figure 14b as a result of the deformation suffered by the sample at the moment of the impact of the dart. Furthermore, a higher resistance of the composites is observed when they are impacted on the side of the reinforcement (Figure 14a,b). When the samples impacted on the injection side are analysed, it is detected that beyond appearance of radial fractures, circumferential fractures occur and that means failure of Mode II. In Figure 15c,d, the formation of the radial fracture and the subsequent circumferential delamination are distinguished.

Similarly, analysing both sides of the impact on PAGF-PA86 samples, it is noticed the fracture begins with radial cracks which then evolve to circumferential ones with a manifest pattern of rigid material (Figure 15). This is due to the high rigidity material of its base, since it is reinforced with short fibre. It could be considered in the failure of Mode III.

One of the reasons for the behaviour of the composites in the impact test could be the effect of delamination. However, because delamination reduces the bending stiffness and allows further deformation to occur, it also has a beneficial effect by limiting stresses that lead to fibre failure. If it were completely removed, there would be a risk of premature fibre failure, which could lead to a more brittle overall response [49,50].

### 3.5. Three-Point Loading Test

**Figure 16:**
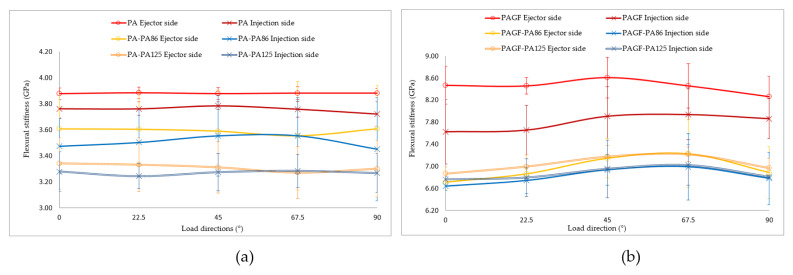
Comparison of flexural test results according to the impact side on the sample between composites and matrix material without reinforcement. (**a**) PA matrix. (**b**) PAGF matrix.

Regarding the monotonic three-point flexural test, the samples are not tested until breakage, thus only their behaviour in elastic mode was registered. The obtained values of the elastic modulus of PA and PAGF matrix are significantly higher than the ones obtained by Nagakura et al. [51], and these results may be due to the fact that PA used in this work is PA66, not PA6. In the Figure 16, it is verified that none of the composites show a positive gain, that is to say, the properties of the composites registered lower flexural stiffness than their respective matrices. This loss of flexural stiffness was more notable for the composites with PAGF matrix. It can be considered that the lower adhesion of the fabric to the PAGF matrix with respect to the PA contributes to the decrease of the flexural stiffness.

## 4. Conclusions and Perspectives

The aim of this research work was to carry out an industrialization approach of a reinforced textile material based on a PA matrix with and without percentage of GF. The results obtained have shown that it is possible to make composites with PA matrix that have improved energy absorption, with better performance than with PA66GF matrix.

To summarize the results obtained, the properties of each of the composites analysed in the different tests performed are listed in Table 11.

After analysing the results, the following conclusions were obtained:

- Regarding the manufacture of the fabric, the one that seems the most industrially viable, taking into account the materials currently available at the market, is the over-stitching technology.
- The fabric placement system is considered critical regarding the manufacturing phase of the composites.
- The proposed fixing system has allowed the fabric to rest on one of the cavities of the mould, in order to avoid problems and favour the consolidation of the part, even being a totally manual system.
- It has been proven that this over-moulding technology induces a peculiar and asymmetric morphological structure in the materials. The analysis of the morphological structure of the composites, by means of reflection microscopy, showed that the low thermal conductivity of the hybrid fabrics induced an asymmetry in the morphological structure of the composites, caused by the lower cooling velocity on the side of the fabric, with respect to the injection side.
- Peeling tests prove that the use of hybrid textiles on the PA matrix allows to improve the adhesion on the matrix-fabric interface. The worst adhesion appears between the hybrid fabrics and PAGF matrix. This is mainly due to the higher viscosity of this matrix and the fact that the GF filler already has its own coupling agents that could be incompatible with the fabric.
- With respect to the impact tests and from the point of view of energy absorption, it can be ensured that the PA matrix composites have a much better behaviour than the GF reinforced PA matrix composites.
- The stiffness of the composites reinforced with PA matrix fabrics can have improvements of up to 5%, however, this is not considered an industrial scale improvement.

## Figures and Tables

**Table 1:** Mechanical and physical properties of selected materials.

Properties	PA	PAGF
Density (g/cm^3^)	1.14	1.41
Young’s modulus (GPa)	1.4	8.30
Bending modulus (MPa)	1210	-
Impact resistance—Charpy-V-notch (J/m)	110	15
Poisson’s ratio	0.41	-

**Table 2:** Characteristics of the fiberglass fabrics.

Characteristics	GF86		GF125	
	Weft	Warp	Weft	Warp
Fibre percentage	49	51	53	47
Fibre type	EC9 34	EC9 34	EC9 34X2	EC9 34X2
Number of threads per cm	12.0	12.5	9.6	9.0

**Table 3:** Characteristics of the PA fabric (Monodur^®^ from Cadisch Precision Meshes) and PA thread (Passat20 from Hicoman).

Characteristics	PA Fabric	PA Thread
Weight (g/m^2^)	110	
Filament diameter (μm)	210	
Mesh opening (μm)	500	
Diameter (mm)		0.50
Linear density (TEX)		179.92
Resistance (N)		108.50
Deformation (%)		29

**Table 4:** Fibre characteristic from 3B Fibreglass.

Characteristics	Fibre
Density (g/cm^3^)	2.62
Linear density (TEX)	1200
Filament diameter (µm)	17

**Table 5:** Design of experiments.

Matrix	Fabric (Reinforcement Material)	Sample Acronym	Number
PA	None	PA	1
PAGF	None	PAGF	2
PA	GF86 (conventional fabric)	PA-86	3
	GF125 (conventional fabric)	PA-125	4
PAGF	GF125 (conventional fabric)	PAGF-86	5
	GF86 (conventional fabric)	PAGF-125	6
PA	PAGF86 (over-stitched fabric)	PA-PA86	7
	PAGF125 (over-stitched fabric)	PA-PA125	8
	GFF (weaved fabric)	PA-F	9
	GFFPA (weaved fabric)	PA-FPA	10
PAGF	PAGF86 (over-stitched fabric)	PAGF-PA86	11
	PAGF125 (over-stitched fabric)	PAGF-PA125	12
	GFF (weaved fabric)	PAGF-F	13
	GFFPA (weaved fabric)	PAGF-FPA	14

**Table 6:** Injection conditions.

Condition	PA	PAGF
Melt temperature (°C)	290	310
Mould temperature (°C)	50	90
Injection speed (m/s)	43	44
Clamping force (ton)	100	100

**Table 7:** Test performed to different composites.

Function to Verify	Test	Quantity
Adhesion	Peeling test	36
Impact strength	Impact test	42
Stiffness	Three-point loading test	30

**Table 8:** DSC test results.

Material	Melting Temperature (Peak) (°C)		
	Experimental		Datasheet
	$\bar{X}$	s	
PA—Matrix	263.87	1.38	262
PAGF—Matrix	263.21	1.51	262
PA66 thread	257.33	0.95	-
PA66 fabric	262.59	0.40	-

**Table 9:** Tensile test results.

Composite	Material	Maximum Force (kN)	
		$\bar{X}$	s
	GF86	0.19	0.03
	GF125	0.42	0.03
	PA66 fabric	0.59	0.04
	PA66 thread	0.08	0.01
11	PAGF86	0.58	0.03
12	PAGF125	0.59	0.02

**Table 10:** Peeling test results.

Composite	Sample	Mean Peel Strength (N/cm)	
		$\bar{X}$	s
3	PA-86	45.33	18.9
4	PA-125	34.66	3055
7	PA-PA86	44	3.46
8	PA-PA125	44.66	9.45
9	PA-F	29.74	6.9
10	PA-FPA	32.89	7.4
5	PAGF-86	17.33	7.02
6	PAGF-125	25.33	5.33
11	PAGF-PA86	26.66	3.05
12	PAGF-PA125	22	3.46
13	PAGF-F	No adhesion	
14	PAGF-FPA	No adhesion	

**Table 11:** Overview of the composites analysed.

	Fabric Manufacturing	Composite Manufacturing	Deformation	Adhesive	Energy Absorption	Stiffness ^1^
PA-PA86	Good	Good	Good	Good	Poor	8%SLM
PA-PA125	Good	Good	Good	Good	Poor	9%SLM
PA-F	Fair	Good	Good	Poor	-	-
PA-FPA	Fair	Good	Good	Poor	-	-
PAGF-PA86	Good	Good	Good	Good	Fair	10%SLM
PAGF-PA125	Good	Good	Good	Good	Fair	20%SLM
PAGF-F	Fair	Good	Good	Poor	-	-
PAGF-FPA	Fair	Good	Good	Poor	-	-

^1^ Legend: %SLM: Percentage Slightly Lower than Matrix.

## Data Availability

Data is contained within the article.
